# Policy absence and school meal provision in Türkiye: a structured narrative review and policy analysis

**DOI:** 10.3389/fnut.2026.1852549

**Published:** 2026-07-23

**Authors:** Seval Bircan Yılmaz Yıldız

**Affiliations:** Strategic Education and Bologna Coordination, Çanakkale Onsekiz Mart University, Çanakkale, Türkiye

**Keywords:** school meals, policy absence, school food environment, child nutrition, policy analysis, social reproduction, Türkiye

## Abstract

**Introduction:**

School meals are increasingly recognized as instruments for supporting child nutrition, educational participation, and social protection. However, limited attention has been given to how the absence of an institutionalized national program can be distinguished from implementation failure, low policy priority, or fragmented provision. This study examines the institutional organization of school food provision in Türkiye.

**Methods:**

A structured narrative review was combined with qualitative policy document analysis. Epidemiological studies, national institutional reports, legislative and regulatory documents, international guidelines, and comparative program evidence were reviewed. Policy documents were assessed across five dimensions: legal and institutional commitment, program coverage and entitlement, financing, implementation responsibility and continuity, and monitoring and accountability.

**Results:**

The reviewed evidence indicates that nutritional vulnerability among children and adolescents in Türkiye includes excess body weight, irregular meal patterns, selected micronutrient-related concerns, and socioeconomic conditions that may constrain access to adequate food. The school food system combines household provision, regulated commercial food services, and publicly financed meals for particular eligible groups. Although these arrangements demonstrate that public intervention exists, the document analysis did not identify a comprehensive national program combining clearly defined entitlement, recurrent financing, designated implementation responsibility, continuity, nutritional standards, and integrated monitoring. The Turkish case is therefore characterized by policy absence at the level of institutionalized national program provision, coexisting with regulatory, targeted, and fragmented interventions.

**Discussion:**

The study does not establish a causal relationship between this institutional configuration and specific nutritional or educational outcomes. It proposes a state-guaranteed, multi-level governance framework combining national financing and standards with decentralized implementation, professional nutritional oversight, participatory monitoring, and independent evaluation. The study contributes an operational framework for analyzing policy absence as an observable institutional condition rather than as the complete absence of public action.

## 1 Introduction

School meal programs occupy an increasingly important position at the intersection of education, public health, and social protection. They may support access to regular and nutritionally adequate food during the school day, while also contributing to school participation, dietary quality, and the reduction of food-related inequalities. Their significance therefore extends beyond the technical delivery of meals. School food policies determine who receives support, under what conditions, through which institutions, and with what standards of quality and accountability (1–3).

Research on school meals has commonly focused on program design, implementation, nutritional outcomes, and educational effects. Less attention has been given to situations in which no comprehensive national program has been institutionalized. In such contexts, food consumed during the school day may be provided by households, purchased through commercial outlets, supplied through targeted administrative arrangements, or obtained through a combination of these mechanisms. The absence of a comprehensive program should not automatically be interpreted as complete governmental inactivity, because regulatory measures and limited forms of provision may still exist.

This distinction creates an important policy-analytical problem. A country may regulate school canteens, provide meals for selected groups, or repeatedly debate universal provision without establishing a nationally coordinated program. Such a configuration may represent policy absence, fragmented provision, low policy priority, or implementation failure, depending on the presence of legal commitment, defined entitlement, recurrent financing, designated responsibility, implementation continuity, and monitoring arrangements. These conditions must be examined empirically rather than inferred from the simple non-existence of a universal program. The institutional organization of school food provision also has potential distributional implications. When access to food during the school day depends substantially on household preparation, individual purchasing power, or eligibility for targeted support, students may experience different conditions of access. These differences do not by themselves demonstrate that a particular policy arrangement causes nutritional or educational inequality. They do, however, raise questions about how responsibility for children's daily nutrition is distributed among households, markets, schools, and public institutions.

School food environments are therefore relevant not only because of the nutritional quality of available products but also because of how food is financed, supplied, regulated, and accessed. Recent international guidance emphasizes that healthy school food environments require a combination of direct food provision, nutritional standards for foods served or sold in schools, and institutional measures that support healthier choices (3). This broader perspective provides a basis for examining school meals as part of educational infrastructure and public governance rather than solely as individual dietary support.

Türkiye provides a particularly relevant case for examining the distinction between fragmented school food provision and policy absence. Although regulations and limited nutrition-related initiatives exist, school food access continues to rely substantially on household provision and market-based school canteens. Whether this institutional configuration should be characterized as policy absence, implementation failure, low policy priority, or fragmented provision requires systematic examination rather than assumption. Accordingly, this study combines a structured narrative review with qualitative policy document analysis to examine nutritional evidence, the institutional organization of school food provision, and the legislative trajectory of school meal initiatives in Türkiye. It addresses the following research questions:

- What does recent national and international evidence indicate about the nutritional risks, dietary behaviors, and school food environments experienced by school-age children and adolescents in Türkiye?
- How have legislative and policy initiatives concerning school meal provision evolved in Türkiye between 2009 and 2026?
- To what extent does the observed institutional configuration meet the criteria for policy absence rather than implementation failure, low policy priority, or fragmented provision?
- What policy design principles derived from international school meal programs and guidelines may inform a feasible, equitable, and sustainable school meal governance model for Türkiye?

## 2 Conceptual framework

### 2.1 Policy absence as an analytical concept

Policy analysis commonly focuses on the design, implementation, and effects of formally adopted policies. However, the absence of an institutionalized policy may also shape the distribution of responsibilities, resources, and risks. In this study, policy absence is treated as an observable institutional condition rather than as a synonym for governmental inactivity or a normative judgement about political intention. It concerns whether a recognized public problem is addressed through a coherent and sustained combination of legal commitment, defined entitlement, recurrent financing, designated implementation responsibility, nutritional standards, and monitoring arrangements.

This definition distinguishes policy absence from several related conditions. Implementation failure occurs when a formally adopted program exists but is not delivered as intended or does not achieve its stated objectives. Low policy priority refers to an established policy that receives limited funding, administrative capacity, or political attention. Fragmented provision refers to local, temporary, pilot, or population-specific initiatives that provide some support without constituting a continuous and nationally coordinated system. Policy absence, by contrast, describes a situation in which these partial measures do not develop into an institutionalized national program with clearly defined rights, responsibilities, resources, and accountability mechanisms.

The concept is particularly relevant to school food provision because the absence of a comprehensive program does not leave an institutionally neutral space. Nutritional responsibility may instead remain with households, be channeled through market-based providers, or be addressed through limited and discontinuous initiatives. The consequences of this configuration cannot be assumed in advance and must be examined through empirical evidence. Accordingly, this study evaluates the Turkish case through the observable policy dimensions specified in Section 3 rather than inferring policy absence solely from the non-existence of a universal school meal program.

This approach is informed by critical policy scholarship that treats both policy action and inaction as socially consequential. Bacchi's (4) problem-representation approach draws attention to how policy arrangements define which conditions are recognized as public problems and which responsibilities remain outside formal intervention. Ball's (5) analysis of market-oriented governance further helps explain how public responsibilities may be reorganized through private provision and household choice. More specifically, scholarship on policy inaction emphasizes that the absence of intervention should be examined through institutional decisions, available policy instruments, and documented patterns of non-adoption rather than being reduced to a lack of political activity. These perspectives provide the conceptual basis for examining whether school nutrition in Türkiye is characterized by policy absence, implementation failure, low priority, or fragmented provision.

### 2.2 Social reproduction and the distribution of nutritional responsibility

Social reproduction theory provides a second analytical perspective for examining school food provision. The concept draws attention to the material and care-related activities through which individuals are sustained and enabled to participate in education, employment, and social life. Feeding children is one such activity. Although it is often treated as a private household responsibility, it is also closely connected to the functioning of educational institutions because students' participation in schooling depends partly on the daily provision of adequate food, care, and physical wellbeing (6, 7). From this perspective, school meal programs may redistribute part of the responsibility for children's daily nutrition from households to public institutions. Such redistribution does not eliminate the role of families, but it may reduce the extent to which access to food during the school day depends on differences in household income, care capacity, and access to nutritious food. In the absence of institutionalized provision, families generally retain primary responsibility for preparing, purchasing, or financing food consumed during the school day.

The effects of this arrangement are unlikely to be evenly distributed. Households differ substantially in economic resources, working conditions, time availability, food security, and access to nutritionally adequate products. Consequently, a school food system that relies primarily on household provision and individual purchasing power may reproduce existing socioeconomic differences within the educational setting. This does not mean that the absence of a school meal program directly causes particular nutritional or educational outcomes. Rather, it identifies a plausible institutional mechanism through which broader inequalities may influence students' everyday access to food. Social reproduction theory is therefore used in this study to interpret how responsibility for school-time nutrition is distributed among households, markets, schools, and public institutions. It complements the policy-absence framework by shifting attention from the formal existence of policy to the social consequences of different responsibility arrangements. The analysis focuses particularly on whether institutional arrangements reduce, maintain, or potentially intensify the dependence of students' food access on household resources.

### 2.3 School food environments and the spatial organization of access

The concept of the school food environment draws attention to the physical, institutional, and commercial conditions through which students encounter food during the school day. Access is shaped not only by the availability of particular products but also by the locations in which food is prepared, distributed, sold, and consumed; the rules governing those locations; and the financial or administrative conditions attached to access.

From this perspective, different food-provision arrangements produce different forms of access. A collectively organized meal program may establish a shared institutional entitlement, whereas reliance on commercial canteens generally requires students to make individual purchases. The presence of food within the school therefore does not necessarily imply equal access to it. Access may vary according to purchasing power, available products, school infrastructure, operating rules, and the extent to which nutritional standards are implemented.

Lefebvre's (8) understanding of space as socially produced provides a limited but useful interpretive lens for examining these arrangements. School space is not treated here as a neutral physical setting. Rather, kitchens, dining areas, canteens, classrooms, and food-distribution points reflect institutional decisions about how food is organized and who can access it under particular conditions. A school organized primarily around commercial food sales may structure access differently from one in which meals are provided through a publicly financed and collectively administered system.

The present study does not include school observations, spatial mapping, or direct evidence of students' lived experiences. The spatial perspective is therefore used cautiously to interpret the institutional organization of food access rather than to make claims about how individual students experience particular school spaces. Its analytical contribution lies in showing that school food provision concerns not only which foods are available, but also how access is materially and institutionally organized within the educational environment.

### 2.4 Biopolitics as a supporting interpretive lens

Biopolitics is used in this study as a supporting interpretive lens rather than as the principal analytical framework. In Foucauldian terms, biopolitical governance concerns the ways in which institutions organize, regulate, and differentiate the conditions that sustain population health and wellbeing. School food provision is relevant to this perspective because access to adequate nutrition forms part of the material conditions through which children's health, development, and participation in education are supported. The biopolitical relevance of school food provision does not lie only in direct state intervention. It may also be observed in the institutional boundaries established between public responsibility, household responsibility, and market provision. Decisions concerning whether food is publicly provided, commercially sold, selectively targeted, or left primarily to families contribute to the organization of children's everyday nutritional conditions. However, these arrangements should not automatically be interpreted as intentional strategies of exclusion or control. Their significance must be assessed through documented institutional structures and observable consequences.

In the Turkish case, the biopolitical perspective is used to interpret how the absence of an institutionalized national school meal program may leave access to food during the school day dependent on differentiated household resources and market-based arrangements. This interpretation does not imply that policy absence directly produces particular health outcomes. Rather, it highlights how institutional decisions concerning the provision or non-provision of essential resources may distribute nutritional risks and protective conditions unevenly across the school-age population. This supporting perspective complements the policy-absence and social reproduction frameworks. The policy-absence framework identifies the institutional characteristics of the school food system, while social reproduction theory examines how nutritional responsibility is distributed among households and public institutions. The biopolitical lens adds attention to the broader governance of children's health-related conditions without replacing the empirical analysis of policy documents and nutritional evidence.

Overall, these perspectives establish a layered analytical framework. Policy absence provides the primary institutional concept; social reproduction explains the distribution of nutritional responsibility; the school food environment directs attention to the material organization of access; and biopolitics supports interpretation of how health-related conditions may be differentially structured across populations. The empirical analysis that follows does not treat these theoretical perspectives as findings in themselves. Instead, they are used to interpret patterns identified through the structured review and policy document analysis.

## 3 Methods

### 3.1 Research design

This study employed a structured narrative review combined with qualitative policy document analysis. Two complementary bodies of evidence were examined. The first consisted of epidemiological, institutional, and research-based evidence concerning the nutritional status, dietary behaviors, socioeconomic conditions, and school food environments of school-age children and adolescents in Türkiye. The second consisted of legislative, regulatory, and policy documents concerning school meal provision and school food governance. International guidelines and evaluations of school meal programs were also examined to contextualize the Turkish case and inform the policy discussion.

The study did not involve primary data collection or new statistical analysis. Its purpose was to identify recurring empirical patterns, examine the institutional characteristics of school food provision, and interpret the findings through the concept of policy absence. Accordingly, the analysis does not establish causal relationships between the absence of an institutionalized national school meal program and specific nutritional, health, or educational outcomes.

### 3.2 Information sources and search strategy

The literature search covered peer-reviewed studies, national epidemiological reports, institutional publications, legislative records, policy documents, and international guidelines relevant to school-age nutrition and school food provision. Peer-reviewed literature was searched in Web of Science Core Collection, Scopus, and PubMed. Google Scholar was used as a supplementary source for citation tracking and for identifying relevant reports that were not indexed in the principal bibliographic databases.

The search combined terms relating to school food provision, child and adolescent nutrition, socioeconomic inequality, and the Turkish context. The English-language search terms included combinations of “school meal,” “school feeding,” “school food environment,” “school canteen,” “child nutrition,” “adolescent nutrition,” “meal skipping,” “food insecurity,” “micronutrient deficiency,” “overweight,” “obesity,” “education,” “Türkiye,” and “Turkey.” Turkish-language searches used corresponding terms, including “okul yemegi,” “okul beslenmesi,” “ücretsiz okul yemegi,” “okul kantini,” “çocuk beslenmesi,” “ergen beslenmesi,” “ögün atlama,” “gida güvencesizligi,” “mikrobesin eksikligi,” “obezite,” “çocuk yoksullugu,” and “Türkiye.”

Official and institutional searches were conducted through the websites and document repositories of the Turkish Ministry of Health, the Ministry of National Education, the Turkish Statistical Institute, and the Grand National Assembly of Türkiye. International evidence and policy guidance were obtained from the World Health Organization, the Food and Agriculture Organization, the World Food Program, and UNICEF. Legislative searches included parliamentary questions, legislative proposals, committee documents, budget records, and other official records concerning school meal provision and child nutrition.

The search covered documents published in Turkish or English. Priority was given to epidemiological and policy evidence published between 2016 and 2026. Earlier studies were retained when they represented foundational theoretical contributions, nationally significant datasets, or frequently cited evidence for which no more recent equivalent was available. The final search was completed on April 2026.

### 3.3 Eligibility criteria

Sources were eligible for inclusion when they met at least one of the following criteria: (a) they reported empirical evidence concerning the nutritional status, dietary behaviors, food insecurity, meal patterns, or school food environments of school-age children or adolescents in Türkiye; (b) they constituted official national statistics, epidemiological surveys, institutional reports, regulations, legislative proposals, parliamentary questions, budget records, or other policy documents directly related to school nutrition and school meal provision in Türkiye; or (c) they presented international guidelines, program evaluations, or comparative evidence relevant to the design, implementation, governance, or outcomes of school meal programs.

For the epidemiological and school nutrition evidence, priority was given to peer-reviewed studies and nationally representative or large-scale institutional reports published between 2016 and 2026. Earlier sources were retained only when they provided foundational theoretical concepts, nationally significant baseline data, or evidence for which no sufficiently comparable recent source was identified. The legislative and policy document analysis covered the period from 2009 to 2026, corresponding to the earliest verified parliamentary initiative identified in the document corpus. Documents were included in Turkish or English. Sources concerning children and adolescents enrolled in primary, lower-secondary, or upper-secondary education were eligible. Studies focusing exclusively on preschool children, university students, adults, or clinical populations were excluded unless they contained separately reported findings directly relevant to school-age children. Publications were also excluded when they did not identify their data source or methodology, addressed nutrition without a clear connection to children, adolescents, schools, or education policy, or duplicated information reported in a more complete or authoritative source.

For international program evidence, studies and institutional reports were included when they examined identifiable school meal or school food environment interventions and reported information on program design, implementation, nutritional outcomes, food security, school participation, dietary quality, equity, governance, or sustainability (9). Descriptive references to foreign programs without sufficient information on their institutional structure or outcomes were not used as primary evidence.

### 3.4 Document corpus and data extraction

The final document corpus was organized into four source categories: (a) epidemiological and nutritional evidence concerning school-age children and adolescents in Türkiye; (b) national institutional and statistical reports; (c) legislative, regulatory, and policy documents concerning school food provision; and (d) international guidelines, program evaluations, and comparative studies of school meal systems. A structured data-extraction form was used to ensure consistency across the different types of evidence. For epidemiological and nutritional sources, the extracted information included publication year, data-collection year, study design, geographical scope, sample characteristics, age group, nutritional or dietary indicator, reported result, and study limitations. Particular attention was paid to whether the evidence was nationally representative, regionally based, or derived from a specific clinical or school sample. For legislative and policy documents, the extracted information included the date and type of document, responsible institution or political actor, stated policy problem, proposed intervention, intended population, institutional responsibility, financing arrangements, implementation status, and reported outcome. Parliamentary initiatives were additionally recorded according to proposal or question number, legislative period, submission date, and final status wherever such information was available. For international school meal programs and guidelines, data were extracted on program coverage, entitlement criteria, governance structure, financing, food-provision model, nutritional standards, monitoring mechanisms, local procurement arrangements, and reported nutritional, educational, equity, or sustainability outcomes. These international sources were used to contextualize the Turkish case and identify potentially transferable policy design principles rather than to rank national systems.

The extracted information was entered into a document-analysis matrix and compared across source categories. Numerical findings were reported as presented in the original sources; no recalculation, pooling, or meta-analysis was conducted. When estimates differed across sources, differences in age group, study year, sampling design, and geographical scope were considered before the findings were interpreted.

### 3.5 Analytical framework

The policy documents were analyzed using a five-part framework developed to distinguish policy absence from implementation failure, low policy priority, and fragmented provision. The framework examined: (1) legal and institutional commitment, (2) program coverage and entitlement, (3) financing and resource allocation, (4) implementation responsibility and continuity, and (5) monitoring, nutritional standards, and accountability.

First, legal and institutional commitment referred to the existence of binding legislation, formal policy instruments, or an officially designated national program concerning school meal provision. Second, program coverage and entitlement concerned whether access to school meals was universal, targeted to clearly defined groups, limited to particular locations, or dependent on temporary administrative decisions. Third, financing and resource allocation referred to the existence of a recurrent and identifiable public budget for school meal provision. Fourth, implementation responsibility and continuity concerned whether responsibility was assigned to a clearly identified institution and whether provision was sustained over time rather than delivered through short-term, pilot, or fragmented initiatives. Fifth, monitoring, nutritional standards, and accountability referred to the existence of formal mechanisms for menu standards, food safety, program monitoring, equity assessment, and institutional accountability.

Within this framework, policy absence was not defined as the total absence of all state activity related to child nutrition. Instead, it referred to the sustained absence of an institutionalized, nationally coordinated, and publicly financed school meal program with clearly defined entitlement, implementation responsibility, nutritional standards, and monitoring arrangements. This distinction was necessary because Türkiye has introduced various school food regulations and limited nutrition-related initiatives, but these measures do not necessarily constitute a comprehensive national school meal system.

Implementation failure was distinguished from policy absence by the prior existence of a formally adopted program whose stated objectives were not achieved in practice. Low policy priority referred to a formally recognized and institutionally established policy that received limited political attention, funding, or administrative capacity. Fragmented provision referred to the presence of partial, localized, temporary, or population-specific initiatives that did not amount to a continuous and nationally coordinated system. Each legislative and policy document was examined against the five analytical dimensions. The analysis focused on the observable institutional characteristics of the policy environment rather than on attributing political intention. The classification of the Turkish case was therefore based on the presence or absence of identifiable legal, financial, administrative, implementation, and monitoring arrangements.

Table 1 summarizes the operational criteria used to distinguish policy absence from implementation failure, low policy priority, fragmented provision, and institutionalized provision.

**Table 1:** Analytical criteria used to distinguish policy absence from related policy conditions.

Policy condition	Observable characteristics
Policy absence	No institutionalized national program; no clearly defined entitlement; no recurrent dedicated financing; no designated implementation structure; no comprehensive monitoring system
Implementation failure	A formally adopted program exists, but implementation, coverage, quality, or outcomes do not correspond to stated objectives
Low policy priority	A formal policy exists, but it receives limited funding, political attention, administrative capacity, or institutional support
Fragmented provision	Temporary, local, targeted, pilot, or population-specific initiatives exist without forming a continuous national system
Institutionalized provision	Binding framework, defined entitlement, recurrent financing, designated responsibility, implementation continuity, nutritional standards, and monitoring mechanisms are present

### 3.6 Evidence synthesis and interpretation

The analysis proceeded in three distinct stages. First, empirical evidence was synthesized descriptively to identify recurring patterns in nutritional status, dietary behaviors, socioeconomic vulnerability, and school food provision. Findings were reported according to the populations, age groups, data-collection periods, and geographical scope specified in the original sources. Evidence derived from nationally representative reports was distinguished from findings based on regional, school-based, or clinical samples.

Second, the legislative and policy documents were examined using the five-part analytical framework described above. This stage focused on observable policy characteristics, including legal commitment, program coverage, financing, implementation responsibility, continuity, nutritional standards, monitoring, and accountability. The analysis did not infer political motivations that were not explicitly documented. Instead, it identified recurring institutional patterns across the reviewed policy and legislative records.

Third, the empirical and policy findings were interpreted through the concepts of policy absence, social reproduction, and the institutional organization of school food environments. At this stage, a clear distinction was maintained between empirical evidence, theoretical interpretation, and policy implications. Empirical evidence referred to findings directly reported in the included sources. Theoretical interpretation concerned the possible social and institutional meaning of the observed patterns. Policy implications referred to recommendations derived from the combined evidence and comparative program literature.

Because the included sources differed in study design, population, age group, measurement, and reporting period, the findings were not statistically pooled. Convergence across independent sources was treated as strengthening the credibility of an identified pattern, while contradictory or non-comparable findings were reported with appropriate qualification. Accordingly, terms such as “suggests,” “indicates,” “may contribute to,” and “is consistent with” were used where the evidence did not support causal conclusions.

### 3.7 Methodological limitations and ethical considerations

Several methodological limitations should be considered when interpreting the findings. First, the study relied exclusively on secondary data and document analysis and therefore did not directly examine students' experiences, household decision-making, school-level implementation practices, or the perspectives of policymakers and program administrators. The analysis identifies recurring patterns and institutional conditions but cannot establish causal relationships between the absence of an institutionalized national school meal program and specific nutritional, health, or educational outcomes.

Second, the included epidemiological and nutritional sources differed in their sampling designs, age groups, geographical coverage, measurement procedures, and data-collection periods. Consequently, the reported estimates were not treated as directly comparable unless their methodological characteristics were sufficiently similar. Particular caution was exercised when interpreting regional, school-based, or clinical findings alongside nationally representative evidence.

Third, documentary evidence may not fully capture informal administrative practices, local initiatives, or implementation processes that were not recorded in publicly accessible sources. Legislative proposals and parliamentary debates indicate policy attention and institutional responses, but they do not by themselves provide evidence of program effectiveness or political intention. The analysis therefore focused on formally documented legal, financial, administrative, and monitoring arrangements.

Fourth, although a structured search and data-extraction procedure was used, source selection and interpretation remain subject to researcher judgement. To improve transparency, the search terms, eligibility criteria, analytical categories, and included documents were recorded systematically, and the principal empirical evidence and selected verified legislative and policy documents are summarized in Tables 2 and 3, respectively. Priority was given to official, peer-reviewed, and methodologically transparent sources, while uncertain or non-verifiable claims were excluded from the synthesis.

**Table 2:** Selected nutritional, socioeconomic, and school food environment indicators for children and adolescents in Türkiye.

Domain	Indicator	Population and age group	Data-collection year	Geographical and methodological scope	Reported finding	Source	Relevance to the analysis
Anthropometric status	Overweight and obesity	Second-grade primary-school students, predominantly aged 7–8 years	2022	National school-based surveillance using standardized anthropometric measurements	12.5% were classified as overweight and 9.9% as obese	Ministry of Health (11), COSI-TUR 2022	Provides recent national evidence for younger primary-school children; the estimates should not be extrapolated to older age groups
Anthropometric status	Thinness and stunting	Second-grade primary-school students, predominantly aged 7–8 years	2022	National school-based surveillance using standardized anthropometric measurements	3.6% were classified as thin and 1.4% as stunted	Ministry of Health (27), COSI-TUR 2022	Indicates that residual forms of undernutrition coexist with excess body weight
Anthropometric status	Overweight and obesity among lower-secondary students	Students aged 10–14 years	2021–2022 academic year	National administrative physical-fitness measurements covering approximately 45.2% of enrolled lower-secondary students	20.2% were classified as overweight and 10.8% as obese; 4.3% were classified as thin or severely thin	Ministry of Health (28)	Extends the evidence beyond the younger COSI population, but incomplete participation limits direct population-level interpretation
Anthropometric status	Overweight and obesity among upper-secondary students	Students aged 15–18 years	2021–2022 academic year	National administrative physical-fitness measurements covering approximately 18.4% of enrolled upper-secondary students	14.5% were classified as overweight and 5.9% as obese; 3.7% were classified as thin or severely thin	Ministry of Health (28)	Provides evidence for adolescents while requiring caution because participation did not cover the entire enrolled population
Dietary behaviors	Breakfast consumption	Adolescents aged 15–18 years	2017	National cross-sectional nutrition and health survey	The reported frequency of eating breakfast was 67.6% overall, 72.9% among males and 62.3% among females	Ministry of Health (11), TNHS 2017	Indicates irregular breakfast consumption among a substantial proportion of older adolescents; it does not establish an effect of school meal policy
Dietary behaviors	Daily consumption of selected energy-dense foods and drinks	Children and adolescents aged 15–17 years	2022	Nationwide household survey; consumption reported by mothers or primary caregivers	36.2% were reported to consume sweets daily, 25.2% chips or crackers daily, and 20.8% sugar-containing soft drinks daily	TurkStat (29), Türkiye Child Survey 2022	Provides evidence on dietary quality among older children, although the measures concern reported frequency rather than quantities consumed
Dietary quality and selected micronutrient-related concerns	Intake of iron, zinc, and calcium	Adolescents aged 15–18 years	2017	National cross-sectional nutrition and health survey based on reported dietary intake	Reported intakes of iron, zinc and calcium were below recommended daily levels; consumption of meat and milk products was also reported to be low in this age group	Ministry of Health (11), TNHS 2017; Ministry of Health (27), COSI-TUR 2022	Identifies potential nutrient-intake inadequacy but should not be interpreted as biochemical evidence of micronutrient deficiency
Socioeconomic context	Risk of poverty or social exclusion	Children aged 0–17 years	2025	National official poverty and living-conditions statistics	36.8% of children were at risk of poverty or social exclusion	TurkStat (14)	Indicates substantial socioeconomic vulnerability; it is not a direct measure of food insecurity or malnutrition
Socioeconomic context	Food and non-alcoholic beverage price change	General population	December 2025	National consumer price index	Prices for food and non-alcoholic beverages increased by 28.31% compared with December 2024	TurkStat (15)	Provides contextual evidence of food-affordability pressure but does not directly measure household food access
School food environment	Regulation of school food businesses and products	Students attending public and private educational institutions	2013–2025 regulatory framework	National regulations, ministerial circulars, food-product criteria and inspection documents	Formal requirements cover hygiene, food safety, products sold in school food businesses, product labeling and inspection	Ministry of Agriculture and Forestry (30–32); Ministry of National Education (33, 34)	Demonstrates that the school food environment is regulated and that the case does not represent complete governmental inactivity
School food environment	Targeted publicly financed meal provision	Students covered by transported education and other specified eligibility arrangements	2024–2025 academic year	National administrative provision reported by the Ministry of National Education	902,704 students reportedly received free lunch through targeted administrative arrangements	Ministry of National Education (35)	Demonstrates substantial but eligibility-based public provision rather than generally defined entitlement
School food environment	Universal or generally defined school meal entitlement	Students in compulsory education	Document search updated through June 2026	National legislative and policy document corpus	No verified binding instrument established a recurrent publicly financed school meal entitlement applying to all students	Present document analysis	Supports the classification of policy absence at the level of a comprehensive institutionalized national program
School food environment	Overall institutional structure of provision	School-age population	Document search updated through June 2026	Synthesis of national regulations, administrative arrangements, and legislative records	Household provision, regulated commercial food services and targeted publicly financed meals coexist	Present document synthesis	Indicates a differentiated and fragmented institutional arrangement rather than either complete state inactivity or universal program provision

Findings are reported according to the age groups, data-collection periods, geographical coverage, participation rates, and methodological characteristics specified in the original sources. Estimates derived from different populations and study designs should not be interpreted as directly comparable. Administrative physical-fitness measurements did not cover all enrolled students and are therefore distinguished from national survey evidence. Dietary iron, zinc, and calcium findings concern reported intake relative to recommended levels and do not constitute biochemical diagnoses of deficiency. Policy-related entries are based on the official documents included in the study corpus.

**Table 3:** Selected verified legislative and policy milestones concerning school food provision in Türkiye, 2012–2025.

Year and date	Instrument/document	Initiating authority or actor	Main objective and target population	Financing or implementation arrangement	Verified status	Official source
25 March 2012	Council of Ministers Decision No. 2012/2957: School Milk Program	Council of Ministers; implemented jointly by the relevant ministries	Distribution of milk to preschool and primary-school pupils to support balanced nutrition and milk-consumption habits	Centrally organized interministerial Program; limited to a specified food product and age groups	Implemented as a national product-specific Program; not a full school meal entitlement	Official Gazette No. 28244; Ministry of Agriculture and Forestry
17 December 2015	Bill No. 2/513 amending the National Education Basic Law	CHP parliamentary group; first signatories included Özgür Özel, Levent Gök, and Engin Altay	Full-day education and free lunch for students and personnel in formal educational institutions	Proposed public provision; detailed financing arrangements were not institutionalized	Became void at the end of the legislative period	Grand National Assembly of Türkiye
18 July 2018	Bill No. 2/767 amending the National Education Basic Law	Serkan Topal, CHP	Free lunch for students and education employees in all public formal educational institutions	Proposed public provision	Became void at the end of the legislative period	Grand National Assembly of Türkiye
16 May 2022	Bill No. 2/4461 amending the Primary Education and Education Law	Ahmet Kaya, CHP	Introduction of a free lunch Program in public primary schools	Proposed expenditure from an appropriation placed in the Ministry of National Education budget	Became void at the end of the legislative period	Grand National Assembly of Türkiye
2023–2024 academic year	Transported education and free lunch provision	Ministry of National Education	Free lunch for students covered by transported education arrangements and specified eligible groups	Publicly financed targeted provision administered by the Ministry of National Education	Implemented; 1,029,250 students reportedly received free lunch within transported education provision	Ministry of National Education; parliamentary budget records
6 September 2024	Bill No. 2/2426 on one free meal in all schools	Gülüstan Kiliç Koçyigit, DEM Party	Free food and meal support for students in preschool, primary, lower-secondary, and upper-secondary education	Proposed public support Program	Pending before the relevant parliamentary committees	Grand National Assembly of Türkiye
11 October 2024	Bill No. 2/2601 amending the National Education Basic Law	Ali Gökçek, CHP	One free meal each school day for students attending public preschool, primary, and secondary schools	Proposed public provision	Pending before the relevant parliamentary committees	Grand National Assembly of Türkiye
15 October 2024	Bill No. 2/2609 amending the National Education Basic Law	Evrim Karakoz, CHP	Free lunch for students and personnel in formal educational institutions	Proposed financing through the Ministry of National Education budget	Pending before the relevant parliamentary committees	Grand National Assembly of Türkiye
12 November 2024	Bill No. 2/2688 on the provision of school lunches	CHP parliamentary group; first signatories included Suat Özçagdaş, Ali Mahir Başarir, Gökhan Günaydin, and Murat Emir	Free lunch for students in preschool, primary, and secondary educational institutions	Proposed national public provision	Pending before the relevant parliamentary committees	Grand National Assembly of Türkiye
23 September 2025	Bill No. 2/3286 amending the National Education Basic Law	Cem Avşar, CHP	One free daily meal for all students in public preschool, primary, and secondary schools, based on nutritional requirements defined by the Ministry of Health	Proposed public provision	Pending before the relevant parliamentary committees	Grand National Assembly of Türkiye

*CHP, Republican People's Party; DEM, Peoples' Equality and Democracy Party.

The table reports selected verified milestones rather than every parliamentary statement or policy debate concerning school nutrition. “Became void” indicates that a bill was not enacted before the end of the relevant legislative period; it should not be interpreted as a formal parliamentary rejection. The document search was updated through June 2026, but no verified 2026 initiative meeting the inclusion criteria was added to the table.

All data and documents analyzed in this study were obtained from publicly accessible sources. No primary data were collected, no individuals were recruited, and no identifiable personal information was used. Ethical approval and informed consent were therefore not required.

## 4 Results

### 4.1 Overview of the evidence

The reviewed evidence was organized into four empirical domains: nutritional status, dietary behaviors and micronutrient-related risks, socioeconomic conditions affecting food access, and the institutional organization of school food provision. The findings are presented descriptively and according to the age groups, data-collection periods, geographical coverage, and methodological characteristics reported in the original sources.

Table 2 summarizes the principal nutritional and policy-relevant indicators identified in the reviewed sources. Because the available evidence differs substantially across age groups, study years, and sampling designs, the table reports the characteristics of each source explicitly and does not combine findings into a single national prevalence estimate. The table distinguishes nationally representative evidence from regional or sample-specific findings and avoids treating estimates derived from different age groups or study designs as directly comparable. The purpose of the synthesis is to identify recurring patterns and evidence gaps rather than to produce pooled prevalence estimates or causal conclusions.

As shown in Table 2, the reviewed evidence covers multiple and methodologically diverse dimensions of child and adolescent nutrition, including anthropometric status, dietary behaviors, selected micronutrient indicators, socioeconomic conditions, and the organization of school food provision. The following sections present these domains separately, with attention to differences in age group, data period, geographical coverage, and source characteristics.

### 4.2 Nutritional status of school-age children and adolescents

The available evidence indicates that children in Türkiye experience a combination of overnutrition and residual forms of undernutrition, although the prevalence and nature of these conditions vary according to age group, sex, study year, and measurement method. The findings should therefore be interpreted by population group rather than as a single national estimate applicable to all school-age children and adolescents. The fourth round of the Childhood Obesity Surveillance Initiative in Türkiye reported that approximately 20%−25% of children aged 7–9 years were overweight or obese, while the prevalence of obesity was approximately 8%−10% (10). These findings provide nationally relevant evidence for younger primary-school children but should not be extrapolated directly to older children or adolescents. The age group covered by the COSI study is therefore stated explicitly in both the text and Table 2.

Evidence from the Turkey Nutrition and Health Survey also indicates that overnutrition coexists with residual indicators of undernutrition among children and adolescents (11). However, estimates reported for different age groups and nutritional indicators are not always directly comparable because of differences in age classification, measurement procedures, and reporting formats. Accordingly, the present review reports each indicator together with its corresponding age group, data-collection period, and source rather than combining findings into an overall prevalence estimate. The evidence is consistent with a double burden of malnutrition in which excess body weight coexists with other forms of nutritional vulnerability. Nevertheless, the available data do not establish that these outcomes result from the absence of a national school meal program. They instead provide the epidemiological context within which the organization of school food provision and the distribution of nutritional responsibility are examined.

### 4.3 Dietary behaviors and micronutrient-related risks

The reviewed evidence indicates that nutritional vulnerability among children and adolescents in Türkiye cannot be assessed solely through body-weight indicators. Meal regularity, dietary diversity, and micronutrient status constitute additional dimensions of nutritional wellbeing. National reports identify irregular meal consumption, including breakfast and lunch skipping, as concerns among particular age groups, although prevalence estimates vary according to age, sex, survey design, and the definition of meal skipping used in each source (11).

Meal skipping is relevant to the school context because a substantial part of children's daily food consumption occurs before or during school hours (12, 13). However, the available evidence does not establish that meal skipping is caused by the absence of a national school meal program. The reported patterns may reflect multiple and interacting factors, including household income, family routines, food availability, individual preferences, school schedules, and access to food within or near schools. Accordingly, meal-skipping findings are treated as indicators of potential school-time nutritional vulnerability rather than as direct evidence of policy effects. The available national and large-scale evidence also identifies concerns relating to selected micronutrients, particularly iron and vitamin D, among children and adolescents in Türkiye (11). Iron deficiency and anemia are relevant because of their established associations with fatigue, attention, cognitive functioning, and physical development. Vitamin D deficiency is also frequently reported, although its prevalence may be influenced by dietary intake, sunlight exposure, age, sex, geographical location, and measurement thresholds.

The micronutrient indicators included in this review were selected on the basis of three criteria: the availability of recent national or large-scale evidence, relevance to child and adolescent growth or functioning, and sufficient methodological information to interpret the reported estimates. The review does not claim to provide a comprehensive account of all micronutrient deficiencies among children and adolescents in Türkiye. Evidence concerning other nutrients, including vitamin B12, folate, iodine, zinc, and calcium, is incorporated only when sufficiently recent and methodologically transparent data are identified.

Overall, the evidence suggests that nutritional risk may coexist with adequate or excessive energy intake. This pattern is consistent with the concept of hidden hunger, in which micronutrient inadequacy may occur without visible underweight. Nevertheless, the reviewed evidence describes nutritional conditions and associations; it does not establish that these conditions result directly from the organization of school food provision.

### 4.4 Socioeconomic conditions affecting food access

Socioeconomic conditions form an important part of the context in which children and adolescents access food. According to the most recent official child statistics, 36.8% of the child population in Türkiye was at risk of poverty or social exclusion in 2025, compared with 27.9% of the total population (14). The indicator combines income-related poverty risk with material and social deprivation and low work intensity; it should therefore not be interpreted as a direct measure of child hunger or nutritional deficiency. Nevertheless, it identifies a substantial population experiencing conditions that may constrain household access to adequate material resources.

Food prices constitute a further contextual pressure. Official consumer price statistics indicate that prices in the food and non-alcoholic beverages category increased by 28.31% annually in December 2025 (15). Because this figure represents the change recorded for a specific 12-month period, it is not used here as a general estimate for the entire study period. Rather, it illustrates the continuing volatility and affordability pressures surrounding household food expenditure. Existing research indicates that households facing economic constraints may prioritize caloric sufficiency, price, and product durability over dietary diversity and nutrient density (16). The extent and form of these trade-offs vary across households and cannot be inferred directly from national poverty or inflation statistics. Nevertheless, high exposure to poverty or social exclusion, combined with sustained food-price pressures, may limit the capacity of some households to provide nutritionally adequate and regular food during the school day.

The evidence reviewed in this study does not establish that household income directly determines children's nutritional outcomes. Nutritional conditions are shaped by multiple factors, including household resources, food prices, parental time, dietary preferences, local food availability, health conditions, and institutional support. Socioeconomic indicators are therefore interpreted as contextual determinants of food access rather than as direct evidence of individual dietary intake or health outcomes.

### 4.5 Organization of the school food environment

The documentary evidence indicates that school food provision in Türkiye is regulated but institutionally differentiated. National regulations address the hygiene, safety, nutritional characteristics, and inspection of foods sold or served through school canteens, cafeterias, dining facilities, kiosks, and other food businesses operating within educational institutions. These measures demonstrate that the school food environment is not an unregulated policy area. The regulatory framework includes hygiene requirements for school canteens, rules concerning the foods and beverages that may be sold in educational institutions, product approval and school-food labeling procedures, and institutional inspection mechanisms. The principal focus of these measures is the safety and nutritional suitability of food available through school-based food businesses. They regulate the conditions under which food is sold or served but do not, by themselves, establish an individual entitlement to a publicly financed meal for every student.

Publicly supported meal provision also exists for particular student groups. Current administrative and procurement documents provide free meals for eligible students within the transported education system. Such arrangements demonstrate the presence of targeted institutional provision and should not be characterized as a complete absence of state involvement. However, eligibility is linked to specified administrative categories rather than to a universal entitlement applying to all students enrolled in compulsory education.

The reviewed documents therefore indicate the coexistence of three principal arrangements: regulated commercial food provision within schools, household-provided food, and publicly financed meals for particular eligible groups. These arrangements differ in their conditions of access, financing, and institutional responsibility. The available documentary evidence does not establish the relative proportion of students relying on each arrangement, and the present study therefore avoids describing the canteen model as the exclusive or quantitatively predominant source of school-time food without nationally representative evidence. International profiling published by the Food and Agriculture Organization reported that Türkiye did not have a comprehensive national school meal program as of 2022. Because this assessment predates some of the most recent administrative documents, it is treated as historical contextual evidence rather than as sufficient proof of the current policy configuration. The classification of the Turkish case is therefore based on the combined assessment of current regulatory, financing, coverage, implementation, and monitoring arrangements presented in the policy analysis.

## 5 Discussion

### 5.1 Interpreting the Turkish case through policy absence

The findings do not support the claim that school nutrition in Türkiye is characterized by a complete absence of public intervention. National authorities regulate foods sold or served in schools, establish hygiene and food-safety requirements, and finance meal provision for particular eligible groups. The relevant policy environment is therefore better understood as a combination of regulation, targeted provision, household responsibility, and market-based access. Nevertheless, the reviewed documents did not identify an institutionalized national school meal program combining a generally defined entitlement, recurrent and dedicated public financing, clearly designated implementation responsibility, continuity across school levels, nationally applicable meal standards, and comprehensive monitoring arrangements. When assessed against the five-part analytical framework, the Turkish case therefore displays policy absence at the level of comprehensive national program provision, alongside fragmented and targeted forms of intervention.

This formulation distinguishes the absence of an institutionalized program from the absence of all policy activity. Existing regulations primarily govern the safety and nutritional characteristics of food businesses operating within schools, while targeted meal arrangements apply to defined administrative or socioeconomic categories. These interventions may address important needs, but they do not constitute a common school meal entitlement available to the broader student population. The distinction between policy absence and implementation failure is also important. Implementation failure would require the prior adoption of a comprehensive national school meal program whose objectives were not achieved in practice. The reviewed corpus did not identify such a program. Similarly, the case cannot be described solely as low policy priority because no fully institutionalized national program was identified to which insufficient funding or administrative capacity could be attributed. The evidence is more consistent with the continued coexistence of partial provision and the non-adoption of a comprehensive national framework.

From a social reproduction perspective, this institutional configuration leaves a substantial share of responsibility for school-time nutrition with households and individual students. Food may be brought from home, purchased within or around the school, or provided through targeted public arrangements. Because households differ in income, time, food security, and care capacity, reliance on these differentiated arrangements may reproduce unequal conditions of access during the school day. However, the present analysis does not establish the extent of such inequality at the student level, and direct evidence from students, families, and schools would be required to assess it empirically.

The Turkish case therefore illustrates how policy absence may coexist with substantial regulatory activity. The central issue is not whether the state acts at all, but whether existing activities form a coherent, rights-based, adequately financed, and continuously monitored system of school meal provision. This interpretation avoids treating policy absence as a purely normative accusation and grounds it instead in observable institutional characteristics.

### 5.2 Legislative and policy trajectory of school meal initiatives

The legislative and policy records indicate that school meal provision has entered the national policy agenda repeatedly rather than remaining entirely unrecognized. Parliamentary questions, legislative proposals, budget discussions, administrative programs, and public policy debates have addressed child nutrition, food access, and the provision of free meals in schools at different points during the period examined. Table 3 presents the verified initiatives identified in the document corpus and records their institutional form, stated objective, target population, financing provisions, and final status (36–45).

The trajectory summarized in Table 3 demonstrates recurring policy attention but limited institutional consolidation. The presence of repeated initiatives indicates that child nutrition and school meal provision have been recognized as public concerns by different political and administrative actors. However, recognition of a problem does not by itself constitute the adoption of a comprehensive policy response. The reviewed initiatives varied considerably in form and scope. Some addressed universal or broadly available free meals, whereas others concerned particular products, student groups, administrative programs, or temporary forms of support. These differences are important because a parliamentary question, a legislative proposal, a budget amendment, and an implemented administrative program do not carry the same legal authority or institutional consequences. The analysis therefore distinguishes agenda-setting activity from binding policy adoption and sustained program implementation.

Across the period examined, the reviewed corpus did not identify a legislative or administrative measure that simultaneously established a nationally coordinated entitlement, recurrent financing, clearly designated implementation responsibility, continuity across school levels, and comprehensive monitoring arrangements. Several initiatives drew attention to child poverty, school-time hunger, and unequal access to food, but they did not develop into an institutionalized national school meal program meeting all five analytical criteria. This pattern should not be interpreted as evidence that every proposal was rejected for the same reason or that a single political intention persisted throughout the period. Legislative outcomes may be shaped by fiscal constraints, agenda competition, administrative capacity, partisan disagreement, institutional jurisdiction, and changing governmental priorities. The documentary evidence permits identification of repeated non-adoption or limited adoption, but it does not establish the motives of individual policymakers unless those motives were explicitly recorded.

The legislative trajectory therefore strengthens the interpretation of the Turkish case as one in which policy absence at the level of comprehensive national provision coexists with policy recognition, regulation, and partial intervention. Repeated agenda entry without institutional consolidation is analytically different from complete policy silence, but it also differs from the implementation failure of an already established universal program.

### 5.3 International evidence on school meal programs

International evidence indicates that school meal programs may contribute to nutritional, educational, and social protection objectives, although their effects vary according to program design, population, baseline nutritional conditions, meal quality, coverage, and implementation capacity. The evidence does not support the assumption that food provision alone automatically produces substantial improvements across all health and educational outcomes. A systematic review and meta-analysis of school feeding interventions in low- and middle-income countries found modest improvements in height and weight over a 12-month period and identified benefits for school attendance in some settings (17). However, effects differed across studies and outcomes, and no consistent improvement was identified for every nutritional or educational indicator. These findings suggest that school meals may support children's participation and physical wellbeing, particularly where nutritional deprivation is present, but program effects should not be generalized across contexts (18, 19).

A more recent Cochrane review similarly concluded that free or subsidized school feeding may produce small improvements in mathematics achievement, school enrolment, and selected physical growth indicators among socioeconomically disadvantaged children. The review found little or no effect on reading achievement and reported uncertainty for several other outcomes because of variation in program design and evidence quality (46). These findings reinforce the need for cautious interpretation and for program evaluation using clearly defined nutritional, educational, equity, and implementation outcomes. Recent global evidence also highlights the distinction between targeted and universal provision. Both approaches may address hunger and nutritional vulnerability, but they differ in coverage, administrative requirements, and potential social consequences. Targeted programs concentrate resources on identified groups, whereas universal programs may reduce eligibility barriers, administrative exclusion, and the risk of stigma associated with means-tested provision. Nevertheless, the relative effectiveness of universal and targeted models remains dependent on institutional context, nutritional standards, financing, and implementation quality (20).

The latest World Health Organization guideline identifies three complementary policy domains for creating healthy school food environments: the direct provision of food in schools, nutritional standards or rules governing foods and beverages served or sold at school, and interventions that modify the food environment to encourage healthier choices (3). This framework indicates that an effective school nutrition policy cannot be reduced to meal distribution alone. It also requires standards for food quality, regulation of commercial provision, supportive school environments, implementation capacity, and continuous monitoring.

International experience therefore provides design principles rather than a single model that can be transferred unchanged to Türkiye. The evidence supports attention to adequate coverage, nutritional quality, sustainable financing, institutional responsibility, monitoring, and equity. It does not establish in advance that the adoption of a national program in Türkiye would produce identical outcomes to those observed elsewhere. Comparative evidence is used here to identify plausible policy mechanisms and institutional options that require adaptation to the Turkish educational, fiscal, administrative, and food-system context.

### 5.4 Lessons from Brazil's national school feeding program

Brazil's National School Feeding Program (Ia Nacional de Alprogramntação Escolar—PNAE) provides a relevant institutional comparator because it combines a nationally guaranteed framework with decentralized implementation. Law No. 11.947/2009 established school feeding for students enrolled in public basic education and linked meal provision to food and nutrition education, healthy dietary practices, local food cultures, and sustainable development (21). The Program therefore treats school meals not solely as targeted social assistance but as an established component of public education (22).

The governance structure distributes responsibilities across different administrative levels. The National Fund for Educational Development (FNDE) provides financial and technical assistance, while states, municipalities, the Federal District, and federal educational institutions organize procurement and service delivery. Federal transfers are calculated according to student enrolment, the number of school days, and nationally defined per-capita rates. This structure combines national financing rules and standards with implementation adapted to subnational and local conditions (23). Professional responsibility and social oversight constitute additional elements of the program. School menus are planned and monitored by qualified nutritionists, taking into account nutritional requirements, local dietary practices, cultural traditions, seasonality, sustainability, and the availability of minimally processed foods. School Feeding Councils monitor food procurement, meal quality, financial implementation, and institutional accountability. These arrangements establish identifiable responsibility for both the nutritional content of meals and the use of public resources.

The program also links school feeding to local food systems through mandatory public procurement from family farming. Law No. 11.947/2009 originally required at least 30% of relevant federal transfers to be used for the direct purchase of products from family farmers. This minimum was increased to 45% from 2026. Local procurement is intended to support access to fresh and culturally appropriate food while connecting school meal expenditure with rural livelihoods and regional economic development (21, 23, 24). The Brazilian model should not, however, be presented as free from implementation difficulties. Decentralized delivery requires substantial administrative, procurement, professional, and monitoring capacity at the subnational level. Research has identified considerable territorial variation in compliance with family-farming procurement requirements and in local implementation capacity (25). PNAE therefore demonstrates both the potential of multi-level governance and the continuing need for central equalization, technical assistance, monitoring, and institutional capacity-building.

The principal lesson for Türkiye is not that the Brazilian system can be transferred unchanged. Rather, PNAE illustrates how several currently separate policy elements may be integrated within a coherent framework: nationally defined entitlement, recurrent central financing, decentralized delivery, professional menu planning, participatory oversight, local procurement, and transparent accountability. These principles inform the proposed Turkish model while requiring adaptation to Türkiye's administrative structure, municipal capacities, school infrastructure, agricultural supply systems, and fiscal conditions.

### 5.5 Toward a state-guaranteed multi-level school meal model

The findings suggest that a viable school meal model for Türkiye should combine a nationally guaranteed framework with decentralized and context-sensitive implementation. The proposed model is therefore defined as state-guaranteed rather than non-state. Central government would retain responsibility for establishing entitlement, minimum nutritional standards, financing principles, monitoring requirements, and mechanisms for reducing territorial inequalities. Municipalities, schools, local producers, universities, health professionals, families, and students would participate in implementation and oversight within this nationally defined framework.

At the national level, the Ministry of National Education, in coordination with the Ministry of Health, the Ministry of Agriculture and Forestry, and relevant social protection institutions, would establish the legal and regulatory framework. This framework would define program objectives, eligible populations, minimum meal standards, food-safety requirements, procurement principles, implementation responsibilities, and reporting obligations. A dedicated and recurrent national budget would be required to prevent the program from depending solely on temporary projects, voluntary contributions, or differences in local fiscal capacity. A mixed financing structure may be appropriate. Central government funding should cover the core cost of entitlement and provide an equalization mechanism for municipalities and regions with limited fiscal or administrative capacity. Municipal resources, social policy budgets, and other public funds may supplement—but should not replace—the national financial guarantee. Such an arrangement would reduce the risk that access and meal quality vary primarily according to municipal wealth.

Municipalities could coordinate food procurement, central or local kitchen facilities, transportation, storage, and distribution where administrative and infrastructural capacity is available. However, decentralized implementation should not imply the transfer of full financial responsibility to local government. National standards, technical assistance, and equalization funding would be necessary to prevent decentralization from reproducing regional inequalities. Schools would be responsible for the daily organization of meal service, recording participation, identifying operational problems, and creating appropriate spaces and schedules for food consumption. School administrators should not independently determine eligibility, nutritional standards, or core financing. Their role would be implementation within a nationally regulated system rather than discretionary provision. Qualified nutritionists and public-health professionals would develop age-appropriate menus, monitor nutritional quality, and adapt meals to health requirements, cultural practices, seasonality, and local food availability. Food-safety inspection should remain under the authority of the relevant public institutions. Universities may contribute through independent monitoring, program evaluation, menu development, implementation research, and the public reporting of nutritional, educational, equity, and sustainability indicators. Local agricultural producers, cooperatives, and other qualified suppliers could participate through transparent public procurement mechanisms. Procurement criteria may give appropriate weight to food safety, nutritional quality, seasonal availability, environmental sustainability, and support for local economies (26). However, local procurement should remain subject to national quality and accountability standards and should not be assumed to be feasible in the same form in every region.

Participatory oversight is also necessary. School meal councils or committees could include representatives of school administrations, teachers, parents, students, nutrition professionals, municipalities, and civil-society organizations. Their role would include monitoring meal quality, accessibility, complaints, procurement transparency, and the experiences of participating students. Such bodies should complement rather than replace formal administrative and financial auditing. The proposed allocation of responsibilities across national, local, school-level, professional, and participatory actors is summarized in Table 4.

**Table 4:** Proposed distribution of responsibilities in a state-guaranteed multi-level school meal model for Türkiye.

Actor	Principal responsibilities
Central government	Legal entitlement, core financing, national standards, equalization funding, program monitoring, and accountability
Ministry of National Education	National coordination, school implementation framework, student participation records, and administrative oversight
Ministry of Health	Nutritional guidance, public-health standards, health monitoring, and technical support
Ministry of Agriculture and Forestry	Food-safety standards, supplier regulation, inspection, and procurement guidance
Municipalities	Procurement, kitchens, logistics, storage, and distribution where capacity permits
Schools	Daily meal organization, suitable time and space, operational reporting, and student feedback
Nutrition professionals	Menu planning, nutritional quality assurance and adaptation to age and health needs
Universities	Independent evaluation, monitoring, implementation research and public reporting
Local producers and cooperatives	Supply of eligible products through transparent procurement procedures
Parents and students	Participation in feedback, oversight, and school meal councils

**Figure 1:**
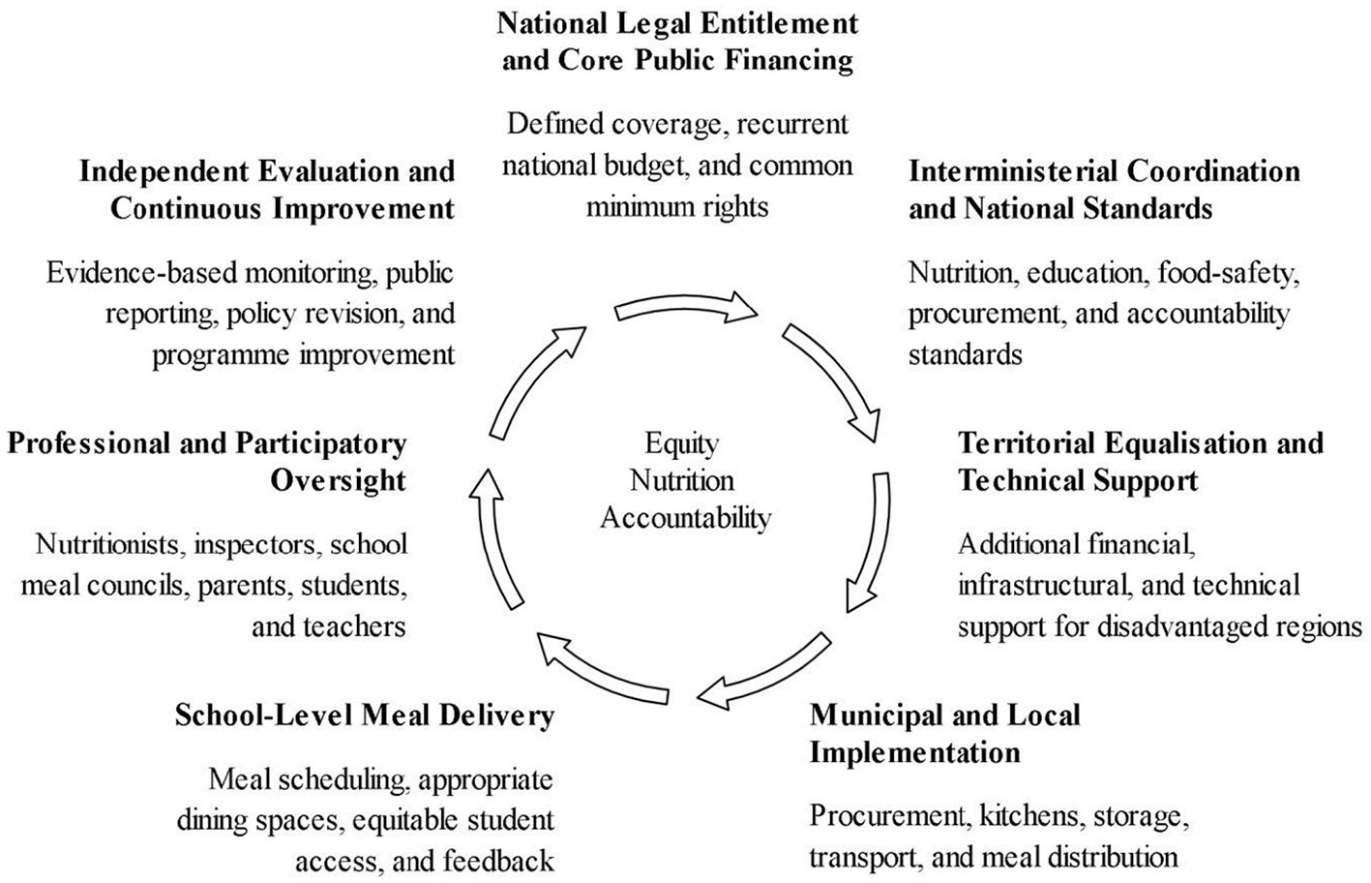
Proposed state-guaranteed multi-level school meal governance model for Türkiye. This figure was generated using Napkin AI and the input prompt has been included in Supplementary File 1.

Implementation could proceed incrementally. Initial phases may prioritize schools serving populations with high levels of socioeconomic vulnerability, while establishing a legally defined pathway toward broader entitlement. Pilot implementation should include predefined evaluation criteria and a clear scaling strategy; otherwise, temporary projects risk reinforcing fragmented provision. Expansion decisions should be based on evidence concerning participation, food quality, student acceptability, operational cost, equity, food waste, and implementation capacity. The proposed model therefore combines national entitlement and financing with decentralized delivery, professional nutritional oversight, local procurement where feasible, participatory monitoring, and independent evaluation. Its purpose is not to prescribe a single operational format for all schools, but to establish common rights, minimum standards, and accountability mechanisms while allowing implementation to respond to local conditions. Figure 1 integrates these institutional responsibilities into a continuous governance cycle connecting national legal and financial guarantees with decentralized implementation, school-level delivery, participatory oversight, independent evaluation, and evidence-based policy improvement.

### 5.6 Theoretical implications

The analysis contributes primarily to the conceptualization of policy absence. The Turkish case demonstrates that policy absence need not involve complete governmental inactivity. Regulatory measures, targeted provision, parliamentary initiatives, and limited administrative programs may coexist with the absence of a comprehensive and institutionalized national program. Policy absence is therefore more accurately understood as a specific institutional configuration rather than as an empty policy space. This distinction also clarifies the relationship between policy absence and fragmented provision. Fragmented interventions may respond to particular needs without establishing common entitlement, recurrent financing, coordinated implementation, or comprehensive accountability. The Turkish case suggests that repeated recognition of a policy problem does not necessarily produce institutional consolidation. The analytical importance of policy absence lies in examining what remains unestablished despite the presence of partial action.

Social reproduction theory helps interpret the distributional implications of this institutional configuration. Where access to food during the school day depends substantially on household preparation, individual purchasing power, or eligibility for targeted support, nutritional responsibility remains unevenly distributed among families, markets, and public institutions. Because households differ in income, time, food security, and care capacity, these arrangements may allow socioeconomic inequalities originating outside school to shape students' conditions of participation within school.

The spatial perspective further indicates that food access is organized through the material and institutional structure of the school environment. Canteens, dining facilities, kitchens, food-distribution arrangements, and the absence or presence of shared meal provision reflect different forms of institutional organization. However, because the present study did not include direct observation or student accounts, it cannot determine how these spaces are experienced by different groups of students. The contribution of the spatial perspective is therefore interpretive rather than empirical.

From a biopolitical perspective, school food policy concerns the institutional organization of conditions that support children's health, development, and educational participation. The findings do not indicate that the absence of a comprehensive program constitutes an intentional strategy to expose particular populations to nutritional risk. Rather, they suggest that decisions concerning public provision, targeted eligibility, market access, and household responsibility may distribute protective conditions unevenly across the student population.

These perspectives show that the significance of school meal policy extends beyond the presence or absence of food provision. It concerns how responsibility, entitlement, resources, and access are institutionally organized. The theoretical contribution of the study therefore lies in connecting policy absence to the everyday distribution of the material conditions that support participation in education, while maintaining a distinction between documented institutional arrangements and their theoretically interpreted consequences.

### 5.7 Limitations and future research

The findings should be interpreted in light of several limitations. First, the study is based on secondary evidence and publicly accessible policy documents. It does not include primary data from students, parents, teachers, school administrators, canteen operators, municipal officials, or national policymakers. Consequently, the analysis cannot determine how school food arrangements are experienced in everyday practice or how different groups respond to existing provision.

Second, the available nutritional evidence is uneven across age groups, indicators, geographical areas, and data-collection periods. Nationally representative information is more readily available for some outcomes and age groups than for others. In particular, recent nationally comparable data concerning older children and adolescents, meal skipping, food insecurity, and micronutrient status remain limited or inconsistently reported. This restricts the possibility of presenting a comprehensive and directly comparable national profile.

Third, the analysis of legislative and policy documents identifies formal initiatives, institutional arrangements, and documented outcomes, but it cannot fully capture informal negotiations, administrative practices, local implementation, or the political reasoning underlying non-adoption. Repeated non-adoption of proposals should therefore not be interpreted as evidence of a single or continuous political intention.

Fourth, the proposed multi-level school meal model is conceptual. Its financial feasibility, administrative requirements, infrastructure needs, public acceptability, and political sustainability have not been empirically tested. The model should therefore be understood as a framework for policy development and evaluation rather than as a fully specified implementation plan.

Future research should include school-level and household-level studies examining how students obtain food during the school day, how access differs across socioeconomic and geographical contexts, and how existing canteen, household-provision, and targeted meal arrangements operate in practice. Qualitative research with students and families would be particularly valuable for understanding experiences of affordability, stigma, food preferences, meal skipping, and participation in school life. Further research should also examine variation across municipalities and regions in school infrastructure, procurement capacity, kitchen facilities, food-service personnel, and access to local agricultural supply chains. Such evidence would help assess whether decentralized implementation could be introduced without reproducing territorial inequalities. Pilot studies of publicly financed school meal provision should incorporate prospective evaluation from the outset. Relevant outcomes should include participation, dietary quality, student acceptability, attendance, concentration, household food expenditure, equity, food safety, operational cost, food waste, and implementation fidelity. Comparative evaluations of universal and targeted approaches would also help determine which model is most appropriate under different fiscal and institutional conditions in Türkiye.

Finally, future policy research should examine the legal, fiscal, and administrative steps required to transform fragmented provision into an institutionalized national framework. This includes analysis of interministerial responsibility, central–local financing arrangements, procurement law, professional staffing, monitoring systems, and mechanisms for student and family participation.

## 6 Conclusion

This study examined the nutritional and institutional context of school food provision in Türkiye through a structured narrative review and qualitative policy document analysis. The reviewed evidence identified three recurring patterns: the coexistence of excess body weight with other forms of nutritional vulnerability; socioeconomic conditions that may constrain some households' access to regular and nutritionally adequate food; and a differentiated school food system combining household provision, regulated commercial food services, and publicly financed meals for selected student groups.

The findings do not indicate a complete absence of public action in school nutrition. National authorities regulate school food businesses and provide meals through particular targeted arrangements. However, the document analysis did not identify a comprehensive national program combining clearly defined entitlement, recurrent public financing, designated implementation responsibility, continuity, nutritional standards, and integrated monitoring. The Turkish case is therefore best characterized as policy absence at the level of institutionalized national program provision, coexisting with regulatory, targeted, and fragmented interventions. The available evidence does not establish that the absence of such a program directly causes particular nutritional, health, or educational outcomes. Nevertheless, continued reliance on household resources, individual purchasing power, and category-based eligibility may leave students with unequal conditions of access to food during the school day. This possibility requires direct investigation through student-, household-, school-, and municipality-level research.

A feasible policy response would require more than meal distribution alone. It would need to combine national legal and financial guarantees with nutritional standards, professional oversight, decentralized implementation, territorial equalization, transparent procurement, participatory monitoring, and independent evaluation. International experience, particularly Brazil's multi-level program, provides useful design principles, but any model would require adaptation to Türkiye's administrative, fiscal, educational, and food-system conditions.

The principal contribution of this study is to conceptualize policy absence as an observable institutional configuration rather than as complete governmental inactivity. By distinguishing comprehensive policy absence from implementation failure, low priority, and fragmented provision, the analysis offers a framework for evaluating how school nutrition responsibilities, resources, and entitlements are organized. Future empirical and pilot research is needed to assess the feasibility, cost, acceptability, and effects of alternative school meal models in Türkiye.

## Data Availability

Official legislation, annual reports, congress meetings were analyzed in this study. Requests to access these datasets should be directed to https://www.tbmm.gov.tr/Yasama/KanunTeklifi/8555889e-2f98-4bbd-bc5c-01931f534a72.
