# Supplementary material for: Policy absence and school meal provision in Türkiye: a structured narrative review and policy analysis
Source: Front Nutr. 2026 Jul 23;13:1852549. doi: 10.3389/fnut.2026.1852549 (PMC13445964; doi:10.3389/fnut.2026.1852549)
Supplement: Supplementary file 1 [file Supplementary_file_1.docx]

**Napkin AI Usage Report**

**Generative AI disclosure:** Figure 1 was generated using Napkin AI, a web-based text-to-visual generation platform provided by Second Layer, Inc. The figure was generated using the web application version available at that time (<https://app.napkin.ai/page/CgoiCHByb2Qtb25lEiwKBFBhZ2UaJDcwODE5ZGZlLWExNzgtNGUyZC1iN2RmLWFiMmJlZjY4OWRkZQ>). Napkin AI did not display a public version number or disclose the specific underlying model used for the individual output. The authors reviewed and edited the generated visual, verified its correspondence with the conceptual framework presented in the manuscript, and take full responsibility for the final figure.

Create a clear, publication-quality academic conceptual figure titled:

“Proposed State-Guaranteed Multi-Level School Meal Governance Model for Türkiye”

The figure should present a vertically organised, cyclical governance model showing how a nationally guaranteed school meal policy moves from legal entitlement and public financing to local implementation, school-level delivery, monitoring, evaluation, and continuous policy improvement.

Use a clean landscape layout with a white background. The figure should be suitable for inclusion in a peer-reviewed academic journal. Use professional typography, simple icons, balanced spacing, thin arrows, and restrained colours. Avoid decorative illustrations, photographs, excessive gradients, and overly bright colours. The figure should appear analytical and institutional rather than promotional.

CENTRAL STRUCTURE

Place a large vertical sequence of seven connected rectangular boxes in the centre of the figure. Use downward arrows between the boxes to indicate the main implementation process.

BOX 1 — NATIONAL GUARANTEE

Title:

National Legal Entitlement and Core Public Financing

Subtitle:

Defined coverage, recurrent national budget, and common minimum rights

Include the following key elements inside or immediately below the box:

• Legal recognition of school meal provision
• Clearly defined student entitlement
• Recurrent and dedicated national funding
• Minimum national service standards
• Protection from dependence on temporary projects or voluntary contributions

This box should visually represent the legal and financial foundation of the entire model. Use an icon suggesting law, government, or public finance.

Draw a downward arrow from Box 1 to Box 2.

BOX 2 — NATIONAL COORDINATION AND STANDARDS

Title:

Interministerial Coordination and National Standards

Subtitle:

Integrated education, nutrition, health, food-safety, and procurement framework

Include:

• Ministry of National Education as the main coordinating authority
• Coordination with the Ministry of Health
• Coordination with the Ministry of Agriculture and Forestry
• Coordination with social protection and public finance institutions
• National nutritional standards
• Age-appropriate menu requirements
• Food-safety rules
• Procurement principles
• Reporting and accountability obligations

Use an icon representing coordinated public institutions or connected government departments.

Draw a downward arrow from Box 2 to Box 3.

BOX 3 — TERRITORIAL EQUALISATION

Title:

Territorial Equalisation and Technical Support

Subtitle:

Additional resources for disadvantaged regions and low-capacity local administrations

Include:

• Equalisation funding for municipalities and regions with limited fiscal capacity
• Technical assistance
• Infrastructure support
• Professional training
• Kitchen and storage investment
• Prevention of territorial inequalities in access and meal quality
• Central support for schools lacking implementation capacity

Emphasise that decentralisation must not allow programme quality to depend on local wealth.

Use an icon representing balanced scales, regional equality, or resource redistribution.

Draw a downward arrow from Box 3 to Box 4.

BOX 4 — LOCAL IMPLEMENTATION

Title:

Municipal and Local Implementation

Subtitle:

Procurement, kitchens, logistics, storage, transport, and distribution

Include:

• Transparent public procurement
• Coordination with local producers and cooperatives
• Central or local kitchen management
• Safe storage and transportation
• Meal distribution logistics
• Adaptation to local food availability and seasonal conditions
• Compliance with national standards
• Municipal implementation without transfer of full financial responsibility from central government

Use an icon representing a municipality, kitchen, food supply chain, or logistics network.

Draw a downward arrow from Box 4 to Box 5.

BOX 5 — SCHOOL-LEVEL DELIVERY

Title:

School-Level Meal Delivery

Subtitle:

Daily organisation of equitable and accessible meal provision

Include:

• Meal scheduling within the school day
• Appropriate dining spaces
• Participation records
• Identification of operational problems
• Student access without stigmatising procedures
• Accommodation of health, disability, allergy, cultural, or dietary requirements
• Student feedback
• Coordination between school administrators, teachers, and food-service personnel

Emphasise that schools implement the programme but do not independently determine entitlement, national standards, or core financing.

Use an icon representing a school, meal tray, or shared dining space.

Draw a downward arrow from Box 5 to Box 6.

BOX 6 — PROFESSIONAL AND PARTICIPATORY OVERSIGHT

Title:

Professional and Participatory Oversight

Subtitle:

Nutrition expertise, inspection, transparency, and stakeholder participation

Divide this box into two connected components:

A. Professional Oversight

• Qualified nutritionists
• Public-health professionals
• Food-safety inspectors
• Age-appropriate menu planning
• Nutritional quality assurance
• Monitoring of portion size and dietary balance
• Compliance with food-safety regulations

B. Participatory Oversight

• Parents
• Students
• Teachers
• School administrators
• Municipal representatives
• Civil-society organisations
• School meal councils or committees
• Complaint and feedback mechanisms
• Monitoring of accessibility, acceptability, transparency, and meal quality

Use icons representing nutrition expertise, inspection, participation, and dialogue.

Draw a downward arrow from Box 6 to Box 7.

BOX 7 — EVALUATION AND IMPROVEMENT

Title:

Independent Evaluation and Continuous Improvement

Subtitle:

Evidence-based monitoring, public reporting, and programme revision

Include the following evaluation indicators:

• Programme coverage
• Student participation
• Nutritional quality
• Equity of access
• Student acceptability
• Food safety
• Attendance
• Concentration and participation in learning
• Household food expenditure
• Operational cost
• Procurement transparency
• Food waste
• Environmental sustainability
• Implementation fidelity
• Regional variation

Also include:

• Independent evaluation by universities or research institutions
• Transparent public reporting
• Use of evidence for programme revision
• Adjustment of financing, standards, procurement, and implementation procedures
• Continuous learning rather than one-time evaluation

Use an icon representing data analysis, research, evaluation, or continuous improvement.

FEEDBACK LOOP

Draw a large curved feedback arrow from Box 7 back to Box 1.

Label this arrow:

Evidence-Based Policy Revision and Continuous Institutional Improvement

The feedback arrow should show that evaluation results influence future legal arrangements, financing levels, national standards, eligibility rules, and implementation design.

LEFT-SIDE ACTOR PANEL

On the left side of the central process, create a vertical panel titled:

National-Level Actors

List:

• Ministry of National Education
• Ministry of Health
• Ministry of Agriculture and Forestry
• Social protection institutions
• Ministry of Treasury and Finance or relevant public finance authorities
• National food-safety and inspection authorities

Connect this panel with thin horizontal lines to Boxes 1, 2, 3, and 7.

The connections should indicate that national institutions are responsible for:

• Legal entitlement
• Core financing
• National standards
• Territorial equalisation
• Monitoring
• Accountability
• Programme revision

RIGHT-SIDE ACTOR PANEL

On the right side of the central process, create a vertical panel titled:

Implementation and Oversight Actors

List:

• Municipalities
• Schools
• Nutritionists and public-health professionals
• Universities and research institutions
• Local producers and agricultural cooperatives
• Parents
• Students
• Teachers
• Civil-society organisations

Connect this panel with thin horizontal lines to Boxes 4, 5, 6, and 7.

The connections should indicate participation in:

• Procurement
• Logistics
• Meal delivery
• Menu planning
• Monitoring
• Feedback
• Independent evaluation
• Local adaptation

CROSS-CUTTING PRINCIPLES

At the bottom of the figure, place a horizontal band titled:

Cross-Cutting Principles

Include the following principles as short labels with small icons:

• Equity
• Universal dignity
• Nutritional adequacy
• Food safety
• Territorial justice
• Transparency
• Accountability
• Student participation
• Cultural appropriateness
• Environmental sustainability
• Local economic development
• Evidence-based decision-making

This band should visually apply to all seven stages of the model.

LOCAL PROCUREMENT COMPONENT

Near Box 4, include a small connected sub-box titled:

Local and Sustainable Procurement Where Feasible

Inside the sub-box, include:

• Family farmers
• Agricultural cooperatives
• Seasonal products
• Fresh and minimally processed foods
• Regional food cultures
• Transparent procurement
• National quality and safety standards

Add a note:

Local procurement should support, but not replace, national responsibility for financing, quality, and equal access.

PHASED IMPLEMENTATION COMPONENT

Near Boxes 3 and 4, include a small sub-box titled:

Phased Implementation and Scaling

Include:

• Initial priority for schools serving highly vulnerable populations
• Predefined evaluation criteria
• Clear pathway towards broader entitlement
• Pilot programmes linked to an explicit scaling strategy
• Prevention of temporary pilots becoming permanent fragmented provision

Use a dotted arrow from this sub-box towards Boxes 4 and 7.

CORE MESSAGE

Place the following sentence in a highlighted box at the top or bottom of the figure:

Decentralised delivery is embedded within a nationally guaranteed system of legal entitlement, core public financing, minimum standards, territorial equalisation, professional oversight, participatory monitoring, and public accountability.

IMPORTANT CONCEPTUAL DISTINCTION

The figure must clearly communicate that:

• The central government guarantees rights, core financing, standards, equalisation, and accountability.
• Municipalities and schools implement the programme within the national framework.
• Local actors participate in delivery and oversight but do not replace state responsibility.
• The model is decentralised in implementation but nationally guaranteed in entitlement and financing.
• The programme is not based on charity, temporary projects, or voluntary contributions.
• Targeted implementation may be used during an initial phase, but the institutional framework should establish a clear pathway towards broader and more equitable entitlement.

VISUAL STYLE

Use a professional academic infographic style.

Recommended layout:

• Landscape orientation
• Central vertical process
• National actors on the left
• Implementation actors on the right
• Cross-cutting principles at the bottom
• Feedback loop from evaluation to national policy
• Clear hierarchy and generous spacing

Use muted institutional colours, for example:

• Dark blue for national legal and financial responsibilities
• Medium blue for coordination and equalisation
• Green for local implementation and procurement
• Warm neutral colour for school-level delivery
• Purple or grey for oversight and evaluation

Do not use more than four or five main colours.

Use consistent rectangular boxes with slightly rounded corners.

Use solid arrows for the main governance process.

Use thin lines for actor connections.

Use dotted arrows for phased implementation or supporting relationships.

Keep all wording legible and avoid overcrowding. If necessary, shorten secondary bullet points while preserving the seven central stages, the two actor panels, the feedback loop, the cross-cutting principles, and the distinction between national guarantee and decentralised implementation.
